# Single time point comparisons in longitudinal randomized controlled trials: power and bias in the presence of missing data

**DOI:** 10.1186/s12874-016-0144-0

**Published:** 2016-04-12

**Authors:** Erin L. Ashbeck, Melanie L. Bell

**Affiliations:** Department of Epidemiology and Biostatistics, University of Arizona, PO Box 245163, Tucson, AZ 85724 USA

**Keywords:** Complete-case, Longitudinal, Mean response profile, Missing data, Mixed model, Power, Repeated measures, *T*-test

## Abstract

**Background:**

The primary analysis in a longitudinal randomized controlled trial is sometimes a comparison of arms at a single time point. While a two-sample *t*-test is often used, missing data are common in longitudinal studies and decreases power by reducing sample size. Mixed models for repeated measures (MMRM) can test treatment effects at specific time points, have been shown to give unbiased estimates in certain missing data contexts, and may be more powerful than a two sample *t*-test.

**Methods:**

We conducted a simulation study to compare the performance of a complete-case *t*-test to a MMRM in terms of power and bias under different missing data mechanisms. Impact of within- and between-person variance, dropout mechanism, and variance-covariance structure were all considered.

**Results:**

While both complete-case *t*-test and MMRM provided unbiased estimation of treatment differences when data were missing completely at random, MMRM yielded an absolute power gain of up to 12 %. The MMRM provided up to 25 % absolute increased power over the *t*-test when data were missing at random, as well as unbiased estimation.

**Conclusions:**

Investigators interested in single time point comparisons should use a MMRM with a contrast to gain power and unbiased estimation of treatment effects instead of a complete-case two sample *t*-test.

**Electronic supplementary material:**

The online version of this article (doi:10.1186/s12874-016-0144-0) contains supplementary material, which is available to authorized users.

## Background

Randomized controlled trials with longitudinal data are sometimes analyzed by comparing an outcome at a single measurement occasion by treatment group, using an independent two-sample *t*-test [1, 2]. When data are complete, the resulting estimated treatment effect and p-value would be the same as if the investigators had used a mixed model for repeated measures (MMRM) to estimate the difference in means (for a continuous outcome) between groups at a given time point [3]. However, if data are missing, results from an MMRM and a *t*-test can differ, as explained below. Missing data in longitudinal trials is common; in a recent review of top medical journals, 95 % of randomized controlled trial publications reported some level of missing data. Though the outcome was collected repeatedly in 79 % of trials, most did not use a model which used all the data, such as a mixed model, opting instead to use only the data available at that time point (e.g., by using a *t*-test) [1]. The implications of this type of analysis may include biased estimation and lower power.

Three missing data mechanisms are described by Rubin [4]. Briefly, when the probability of an observation being missing is not influenced by the values of prior observations, the value of the missing observation, nor other variables, the data are said to be missing completely at random (MCAR). When the probability of a missing observation depends on the value of prior observations but not the value of the missing observation, the data are considered missing at random (MAR). When the probability of missingness depends on the value of the missing (unobserved) value, even after conditioning on observed values, the data are said to be missing not at random (MNAR).

The validity of a *t*-test in a complete-case analysis relies on the assumption that the missing observations are MCAR [5]. It has already been established that in the presence of MCAR or MAR data, an appropriate mixed model will yield unbiased treatment effects on average, as the available data is leveraged in implicit imputation [6].

Baron et al. reported improved power and decreased bias comparing a linear mixed-effects model to complete-case *t*-test analysis of absolute change since baseline, under a single missing data mechanism, in the context of comparing complete-case, last observation carried forward, and multiple imputation [7]. However, to our knowledge, no investigation of power expressly comparing an MMRM to a *t*-test under different missing data mechanisms and missing data types has been published.

Briefly, a MMRM is a means model, also known as a mean response profile analysis, and estimates the mean outcome at each measurement occasion by treatment arm. When an unstructured variance-covariance matrix is specified for the model, the variance of the outcome measure at each observed time and the covariances between each of the repeated measures are all estimated based on the data, without assumption. When a compound symmetric matrix is specified, the variance of the outcome at each observed time is assumed to be equal, and the covariance between any two repeated measures is assumed equal. There is no assumption for the response trajectory over time, thus the risk of bias due to model misspecification is minimal [8]. Further Mallinckrodt et al. reported that MMRM is an appropriate primary analysis for assessing response profiles in a regulatory setting [3, 9].

The primary objective of the simulation study was to compare the power of a mixed model for repeated measures to a complete-case *t*-test, comparing treatment groups at a single time point, in the presence of missing data. The impact of within-person variance and direction of dropout mechanism are considered. The covariance structure used in the analysis was also varied to assess potential power loss under unstructured variance-covariance estimation. The secondary objective was to examine the influence of these factors on estimated treatment effect bias. We show an example using the SF-36 from the Health Evaluation and Linkage to Primary Care (HELP) study, a randomized trial designed to assess the impact of primary medical care on addiction severity [10].

## Methods

### Simulation study

A simulation experiment based on a parallel two-group randomized trial was conducted to investigate power to reject the null hypothesis of no treatment effect, using a complete-case two sample *t*-test and a MMRM at a single time point in a longitudinal study, under different missing data mechanisms, and with different within-person variance, as well as bias of the estimated treatment effect. We used the final time point for analysis.

The outcome was simulated to mimic the Short Form (36) Health Survey (SF-36) norm-based scoring (mean = 50, standard deviation = 10). The SF-36 is a widely used questionnaire that measures health status, consisting of eight scaled scores, each ranging from 0 to 100, where lower scores are indicative of more disability [11].

### Simulation model

Ten thousand datasets were simulated for three different between- and within-person variance scenarios, under a parallel two-group, longitudinal design of four time points, with 100 participants in each arm:

$$ {\mathrm{Y}}_{\mathrm{i}\mathrm{j}}={\upbeta}_1\mathrm{t}1 + {\upbeta}_2\mathrm{t}2 + {\upbeta}_3\mathrm{t}3 + {\upbeta}_4\mathrm{t}4 + {\upbeta}_5{\mathrm{treat}}_{\mathrm{i}}\mathrm{x}\ \mathrm{t}1 + {\upbeta}_6{\mathrm{treat}}_{\mathrm{i}}\mathrm{x}\ \mathrm{t}2 + {\upbeta}_7{\mathrm{treat}}_{\mathrm{i}}\mathrm{x}\ \mathrm{t}3 + {\upbeta}_8{\mathrm{treat}}_{\mathrm{i}}\mathrm{x}\ \mathrm{t}4 + {\upbeta}_{\mathrm{i}} + {\mathrm{e}}_{\mathrm{i}\mathrm{j}} $$

where Y_ij_ = the outcome for the i^th^ subject at the j^th^ time,

i = 1,…,n = 200,

j = 1, 2, 3, 4,

t1 is an indicator variable for time 1 (baseline), and t2 for time 2, t3 for time 3, and t4 is the end-of-study,

treat_i_ = 0 (control), treat_i_ = 1 (treatment),

b_i_ ~ N(0, σ_b_^2^) between-person effects, with σ_b_^2^ between-person variance,

e_ij_ ~ N(0, σ_e_^2^) within-person effects, with σ_e_^2^ within-person variance.

**Fig. 1:**
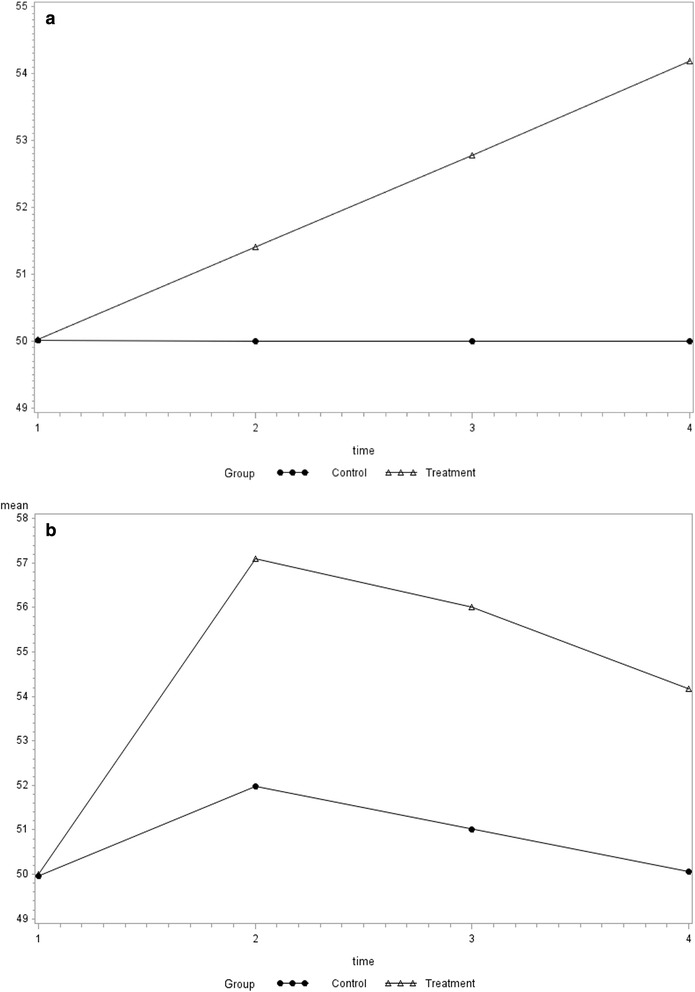
Simulated trajectories of SF-36 Physical Component Summary score. **a**. Linear trajectory. **b**. Non-linear trajectory

The mean baseline SF-36 normed score was set to 50 for both the treatment and control groups. With a sample size of 100 per group, a two sample *t*-test has 80 % power to detect a 4.18 difference between groups, assuming a standard deviation of √110 ≈ 10.4881 in each group (two-sided α = 0.05). The standard deviation was chosen to be similar to the observed standard deviation of the SF-36 Physical Component Summary score from the Health Evaluation and Linkage to Primary Care (HELP) study, a randomized trial designed to assess the impact of primary medical care on addiction severity [10]. The simulations induced an end-of-study treatment difference of 4.18. Two trajectory scenarios were considered, including a treatment effect characterized by a linear trajectory from 50 to 54.18, with no change in the control group, and a non-linear trajectory in both the treatment and control groups, where the treatment effect is initially large and attenuates over time, and the control group experiences a temporary effect (Fig. 1).

A total variance of 110 was assumed for all simulated datasets. Since the between- and within-person variance had the potential to influence comparative performance of the two sample *t*-test and the MMRM, three scenarios were considered. With the total variance fixed at 110 (σ_b_^2^ + σ_e_^2^ = σ^2^ = 110) and assuming compound symmetric variance-covariance structure [12], the first scenario considered was with equal between- and within-person variance of 55, giving ρ = σ_b_^2^/(σ_b_^2^ + σ_e_^2^) = 0.5, based on observed variance components in commonly used psychosocial measures with similar total variance [13]. The second simulated scenario was with between-person variance of 77 and within-person variance of 33, thus ρ = 0.7, which reflects the intuition that repeated observations from a given participant would be more similar than observations from different participants. And finally, in order to consider σ_b_^2^ < σ_e_^2^, a between-person variance of 33 and within-person variance of 77, ρ = 0.3, was simulated. While it may seem counterintuitive that repeated observations from the same participant would have greater variance than observations across participants, it has been reported in practice, and is thus not without precedent [13].

### Missingness type and mechanism

Initially 10,000 complete datasets were simulated under each value of ρ (0.3, 0.5, 0.7), with treatment effect, trajectory, between- and within-person variance as described above. Since the impact of differential dropout on bias has been shown to depend on the directions of dropout mechanisms, ie: different reasons for dropout in each arm [14], we varied the mechanisms as well as considered different scenarios of equal and unequal dropout. We assumed that baseline observations were all complete, and that missing data was monotone (i.e.: participants do not return to the study after dropout). Different missing mechanisms were considered by deleting observations according to the following scenarios

1. *MCAR with equal dropout* of 40 % in each group: Dropout does not depend on health status (Y) at the prior observation or current observation and does not depend on treatment group. [Probability of missingness for participant i at time j (P(M_ij_ = 1)) is based on random sampling].
2. *MAR with unequal dropout* of 30 and 50 % in each group: Participants in the treatment group have a dropout rate of 30 %, while participants in the control group have a dropout rate of 50 %. [P(M_ij_ = 1) = f(Y_i(j-1)_), i.e.: missingness at observation j depends on the value of observation j-1].
  - *One reason for dropout*: This scenario would arise if participants are more likely to dropout when feeling particularly poorly (they stay home), and since the treatment is assumed to have a beneficial effect on health status in these simulations, participants in the control group are more likely to dropout.
  - *Different reasons for dropout:* This scenario would arise if participants are more likely to dropout when feeling particularly poorly (they stay home) or feeling particularly well (take a vacation).
3. *MAR with equal dropout* of 40 % in each group: This scenario could potentially arise via the same mechanism as 2b, where participants drop out for two different reasons, feeling particularly poorly or particularly well, but the dropout rate happens to be the same in each group. [P(M_ij_ = 1) = f(Y_i(j-1)_)].
4. *MNAR with unequal dropout of 30 and 50 % in each group*: Same as 2, except P(M_ij_ = 1) = f(Y_ij_), i.e.: missingness at observation j is dependent on the value of observation j.
  - One reason for dropout.
  - Different reasons for dropout.
5. *MNAR with equal dropout of 40 % in each group:* Same as 3, except P(M_ij_ = 1) = f(Y_ij_).

### Analysis of simulated data

For each sample, subjected to each of the missing mechanisms described above, three analyses were conducted. First, a complete-case two-sample *t*-test was conducted to test the null hypothesis that there is no difference between the group means, using only participants with a non-missing observation at the final time point. The treatment effect was estimated by calculating the difference in group means at the final observation in the complete-case analysis. Second, a mixed model for repeated measures (MMRM) with a contrast was used to estimate the difference between group means at the final time point and test the null hypothesis, assuming a compound symmetric variance-covariance (CS) structure. Additionally, a MMRM was applied similarly, though with unstructured variance-covariance matrix (UN), in order to gauge the potential power loss sustained by estimating more covariance parameters.

### Evaluation of analytical approaches

For each of the three analyses, under the five different missing mechanisms, separately for ρ = 0.3, 0.5, and 0.7, the performance of the analysis was evaluated in terms of power and bias. Specifically, the power of the test was calculated by computing the percentage of *p*-values < 0.05, i.e.: [(Number of p-values <0.05)/10,000] × 100 %. The bias of the estimated difference in group means was assessed based on percent bias, using the simulated treatment effect of 4.18, i.e.: [(estimated difference in group means – 4.18)/4.18] × 100 %. The analyses were initially evaluated in the complete 10,000 datasets (no missing data) in order to confirm the performance and comparability of the analyses in the absence of missing data.

### Example

The HELP study randomized patients with no primary care physician, recruited from a detoxification unit, to multidisciplinary assessment and motivational intervention or usual care, with the goal of linking the patients to primary medical care. The SF-36 was administered at baseline, 6, 12, 18 and 24 months, with substantial missing data due to loss to follow-up. A secondary analysis was conducted to estimate the treatment effect on mental health, assessed with the SF-36 Mental Composite Score (MCS), and compare the estimated treatment difference and corresponding p-value at the 24 month follow-up, using the *t*-test, the MMRM with CS covariance, and the MMRM with UN covariance. Data from the HELP study are publically available (https://www3.amherst.edu/~nhorton/r2/datasets.php).

## Results

### Simulation study

The power and bias estimates were similar for both the linear and non-linear trajectory scenarios, thus only the results for the linear trajectory simulations are described here (Table 1). Results of the non-linear trajectory simulations appear in the supplement (Additional file 1: Table S1). Analysis of the 10,000 complete datasets under each value of ρ confirmed the 80 % planned power, as well as unbiased estimation of the treatment difference at the final time point, using the *t*-test, the MMRM with compound symmetric variance-covariance assumption, and the MMRM with unstructured variance-covariance (Table 1).

**Table 1:** Comparison of *t*-test, mixed model for repeated measures with compound symmetric variance-covariance, and mixed model for repeated measures with unstructured variance-covariance, with respect to bias percent and power; simulation results for linear trajectory

	ρ = 0.7		ρ = 0.5		ρ = 0.3	
	(*N* = 10,000)		(*N* = 10,000)		(*N* = 10,000)	
	Bias %	Power	Bias %	Power	Bias %	Power
Complete						
t-test^a^	0	80	0	80	0	80
MMRM-CS^b^	0	80	0	81	0	80
MMRM-UN^c^	0	80	0	80	0	80
MCAR with equal dropout of 40 % in each group						
*t*-test	0	58	0	58	0	58
MMRM-CS	0	70	0	65	0	61
MMRM-UN	0	70	0	65	0	60
MAR with unequal dropout of 30 % and 50 % in each group, one reason						
*t*-test	−15	44	−11	48	−6	51
MMRM-CS	0	69	0	64	0	60
MMRM-UN	0	69	0	64	0	59
MAR with unequal dropout of 30 % and 50 % in each group, two reasons						
*t*-test	−5	53	−3	53	−2	54
MMRM-CS	0	68	0	63	0	59
MMRM-UN	0	68	0	62	0	59
MAR with equal dropout of 40 % in each group						
*t*-test	−1	57	0	57	−1	56
MMRM-CS	0	69	0	64	0	59
MMRM-UN	0	69	0	64	0	59
MNAR with unequal dropout of 30 % and 50 % in each group, one reason						
*t*-test	−18	42	−16	43	−15	44
MMRM-CS	−6	64	−9	56	−13	49
MMRM-UN	−6	64	−9	56	−13	49
MNAR with unequal dropout of 30 % and 50 % in each group, two reasons						
*t*-test	−7	52	−6	51	−6	51
MMRM-CS	−2	67	−4	60	−5	54
MMRM-UN	−2	67	−4	60	−5	55
MNAR with equal dropout of 40 % in each group						
*t*-test	−3	55	−3	55	−3	55
MMRM-CS	−1	69	−2	63	−2	58
MMRM-UN	−1	69	−2	63	−2	58

^a^Independent two-sample *t*-test for the difference between group means at the final time point

^b^Mixed model for repeated measures, compound symmetric variance-covariance matrix, contrast between group means at the final time point

^c^Mixed model for repeated measures, unstructured variance-covariance matrix, contrast between group means at the final time point

When the data were MCAR with equal dropout of 40 % in each group (scenario 1) the MMRM-CS achieved higher power than the *t*-test, particularly when ρ was higher. As ρ decreased, the power advantage of the MMRM-CS diminished substantially, with a 12 % absolute increase in power when ρ = 0.7, and a 3 % increase in power when ρ = 0.3. Observed loss of power using MMRM-UN was zero or unremarkable. As expected, the estimated treatment difference was unbiased on average.

Under MAR simulation with one reason for dropout (scenario 2a), specifically low value of y at the prior observation, and 30 and 50 % dropout rates by the final time point in the treatment and control groups, respectively, the advantage of the MMRM over the *t*-test became apparent in terms of both power and treatment effect estimation. The power advantage was most pronounced under ρ = 0.7 with a 25 % absolute difference, though the gain was only 9 % under ρ = 0.3. The difference in group means had a -15 % bias under ρ = 0.7, -11 % under ρ = 0.5, and -6 % bias under ρ = 0.3. When data were MAR with unequal dropout, and with two different reasons (scenario 2b) including low or high value of y at the prior observation, a 15 % difference in power gain was observed under ρ = 0.7, though reduced to 5 % under ρ = 0.3. The bias was -5 % for the *t*-test, smaller than when participants dropped out only due to low values of y, while the MMRM continued to provide unbiased estimation of the treatment difference.

While data MNAR is known to present a challenge for estimation even when a MMRM is used, we wanted to evaluate the magnitude of bias and potential power gain under the current missing mechanism scenarios. The difference in bias percent between the complete-case *t*-test and the MMRM was notable when ρ = 0.7 and there was one reason for dropout (scenario 4a), with -18 % bias for the complete-case *t*-test and -6 % for the MMRM, with substantial power gain from 42 to 64 %, though any advantage of the MMRM reduced to a negligible difference under ρ = 0.3. Biased estimation limits the utility of the MMRM in the presence of MNAR data, despite the power gain. More detailed reporting of bias is provided in Additional file 1: Table S2 and S3.

Since most investigators make efforts to minimize missing data, particularly for the primary endpoint, we conducted additional simulations for scenarios 1, 2a, and 4a to evaluate comparative performance with only 10–15 % missing data. The results demonstrated a sustained, though modest, advantage of the MMRM when 10–15 % of the data are missing (Additional file 1: Table S4).

### Example

SF-36 MCS data were missing for 46 % (105/228) of participants randomized to usual care, and 36 % (82/225) of participants randomized to intervention at the 24 month follow-up. The mean SF-36 MCS was 2.28 higher in the treatment group than the usual care group, when considering only those participants who completed the study and the two sample *t*-test produced a p-value of 0.1785. The MMRM with CS variance-covariance matrix estimated a treatment effect size of 2.63 and p-value of 0.0911, while the MMRM with UN variance-covariance matrix estimated a treatment effect of 2.69 and p-value of 0.0946. While the difference in mean SF-36 MCS was not significantly different between treatment groups under any of these analyses, the magnitude of the difference in the estimated effect size and p-value between the complete case *t*-test and MMRM could conceivably distinguish a positive vs. negative trial outcome based on the minimally important difference and/or statistical significance.

## Discussion

Our results demonstrate that a substantial gain in power can be achieved by using a MMRM with a contrast to make a single time point comparison, as compared to an independent two-sample *t*-test. The magnitude of the power gain is influenced by the correlation (ρ) among repeated measures within an individual, equivalently characterized by within-person variance and between-person variance, as higher correlation among repeated measures within an individual provides richer information to be leveraged by the MMRM for implicit imputation of missing observations. While the estimated treatment effect at a single time point calculated by taking the difference in the group means is unbiased when data are MCAR, even with modest correlation (ρ = 0.5) among repeated measures, the improved power warrants use of the MMRM over the complete-case *t*-test when data are MCAR.

The estimation advantage of the MMRM when data are MAR has been previously established, as the MMRM provides unbiased estimation when missingness depends on the values of prior observations, while the complete-case *t*-test does not [8]. Further, our simulation study demonstrates the potential power advantage of the MMRM, also contingent on the magnitude of the within-person variance. Biased estimation continues to limit enthusiasm for use of either the MMRM or *t*-test under MNAR mechanisms.

While we anticipated that estimation of an unstructured variance-covariance matrix would lead to decreased power in the MMRM, as compared to estimation of a compound symmetric variance-covariance structure, our simulations did not support our expectation. The two MMRM generally performed identically in terms of power, at least to the reported level of precision in the table. However, the data were simulated under a compound symmetric variance-covariance structure, and neither of the models we considered represented a misspecification of the true structure. Further, a limitation of our observation is that it cannot be generalized to longitudinal studies with more time points, as the number of parameters to be estimated increases quickly with increasing number of time points, with the number of covariance parameters = n x (n + 1)/2, where n is the number of time points [8]. Since all of our simulations involved four measures, we cannot draw conclusions regarding the magnitude of the power differential between MMRM-CS and MMRM-UN when the study involves more occasions for measurement. An additional limitation is that we only simulated two trajectory scenarios, and more complex trajectories might yield different results with respect to the comparative performance of the MMRM and the complete-case *t*-test. As is always the case with simulation studies, the generalizability of the results beyond the specific induced scenarios is uncertain, and varying all potential factors is impossible.

While Baron et al. reported on the bias and power advantage of a linear mixed-effects model over a complete-case *t*-test of change since baseline, they did not consider the impact of between- and within-person variance, or different directions of dropout, both of which we found to have considerable influence on the comparative performance, an important strength of our simulation study.

## Conclusions

Much has been written about the problems of underpowered studies. If a research question cannot be answered due to underpowering time, effort and resources are wasted, and study participants may be exposed to the potential harms of research [15]. Additionally, underpowered studies contribute to a lack of reproducibility (reliability) in research [16]. Using an MMRM instead of a two sample *t*-test should be considered a relatively simple way to gain power. Investigators who consider a single time point comparison to be the primary scientific question of interest should use a MMRM with a contrast to gain power when data are MCAR, and to gain power and unbiased estimation when data are MAR.

### Ethics approval and consent to participate

Not Applicable

### Consent for participation

Not Applicable

### Availability of data and materials

This was a simulation study. Information regarding simulations is provided in Additional files 2 and 3.

## Additional files

Additional file 1: Supplemental Methods and Results. (DOCX 76 kb) Additional file 2: Supplement SAS simulation and analysis ELA20160310. (PDF 17 kb) Additional file 3: Supplement SAS evaluate performance ELA20160310. (PDF 24 kb)

## Abbreviations

CS: compound symmetric
MAR: missing at random
MCAR: missing completely at random
MMRM: mixed model for repeated measures
MNAR: missing not at random
SF-36: Short Form (36) Health Survey
UN: unstructured
