# Supplementary material for: Single time point comparisons in longitudinal randomized controlled trials: power and bias in the presence of missing data
Source: BMC Med Res Methodol. 2016 Apr 12;16:43. doi: 10.1186/s12874-016-0144-0 (PMC4828848; doi:10.1186/s12874-016-0144-0)
Supplement: Additional file 1: — Supplemental Methods and Results. (DOCX 76 kb) [file 12874_2016_144_MOESM1_ESM.docx]

**Supplemental Text**

Missing data mechanisms

MCAR with equal dropout of 40% in each group

Participants were identified for dropout after baseline (time 1) based on simple random sampling, stratified on treatment arm. The 40 participants per arm were then assigned to drop out at time 2, 3, or 4 by drawing from the uniform distribution [U(0,1)], with a third dropping out at time 2, a third dropping out at time 3, and the final third dropping out at time 4.

MAR with one reason for dropout

Let M_ijk_ =1 if the outcome Y_ijk_ is missing for the ith subject at the jth time in the kth group

$$\Pr\left( M_{\mathrm{ijk}}=1 \right)=c\frac{100-y_{i(j-1)k}}{100}$$

where c is set to obtain desired Pr(M_ijk_=1) in each arm at each timepoint


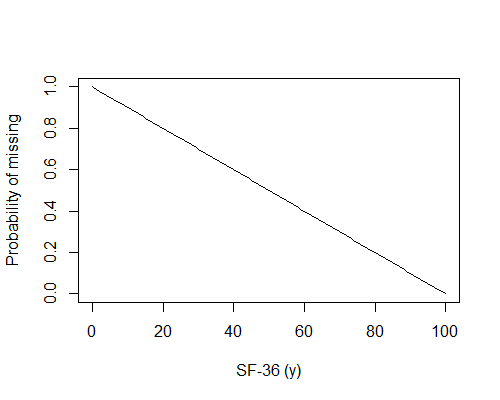


MAR with two reasons for dropout

$\Pr\left( M_{\mathrm{ijk}}=1 \right)=c\left( \frac{55- y_{i\left( j-1 \right)k}}{55} \right)^{2}$
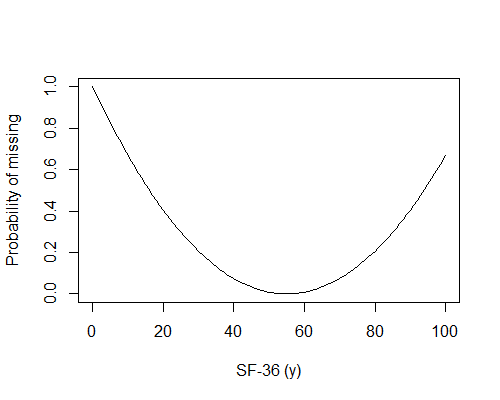


MNAR with one reason for dropout

$$\Pr\left( M_{\mathrm{ijk}}=1 \right)=c\frac{100-y_{\mathrm{ijk}}}{100}$$

MNAR with two reasons for dropout

$$\Pr\left( M_{\mathrm{ijk}}=1 \right)=c\left( \frac{55- y_{\mathrm{ijk}}}{55} \right)^{2}$$

**Supplemental Table 1. Comparison of t-test, mixed model for repeated measures with compound symmetric variance-covariance, and mixed model for repeated measures with unstructured variance-covariance, with respect to bias percent and power; simulation results for non-linear trajectory**

|  |  | **ρ=0.7** | | |  | **ρ=0.5** | | |  | **ρ=0.3** | | |
| --- | --- | --- | --- | --- | --- | --- | --- | --- | --- | --- | --- | --- |
|  |  | (N=10,000) | | |  | (N=10,000) | | |  | (N=10,000) | | |
|  |  | Bias % |  | Power |  | Bias % |  | Power |  | Bias % |  | Power |
| Complete^1^ | |  |  |  |  |  |  |  |  |  |  |  |
|  | t-test^2^ | 0 |  | 80 |  | 0 |  | 80 |  | 0 |  | 80 |
|  | MMRM-CS^3^ | 0 |  | 80 |  | 0 |  | 80 |  | 0 |  | 81 |
|  | MMRM-UN^4^ | 0 |  | 80 |  | 0 |  | 80 |  | 0 |  | 80 |
| MCAR with equal dropout of 40% in each group | | | | |  |  |  |  |  |  |  |  |
|  | t-test | 0 |  | 57 |  | 0 |  | 57 |  | -1 |  | 57 |
|  | MMRM-CS | 0 |  | 70 |  | 0 |  | 64 |  | -1 |  | 60 |
|  | MMRM-UN | 0 |  | 70 |  | 0 |  | 65 |  | -1 |  | 60 |
| MAR with unequal dropout of 30% and 50% in each group, one reason | | | | | | | |  |  |  |  |  |
|  | t-test | -15 |  | 44 |  | -11 |  | 48 |  | -7 |  | 51 |
|  | MMRM-CS | 0 |  | 69 |  | 0 |  | 63 |  | 0 |  | 59 |
|  | MMRM-UN | 0 |  | 69 |  | 0 |  | 63 |  | 0 |  | 59 |
| MAR with unequal dropout of 30% and 50% in each group, two reasons | | | | | | | | |  |  |  |  |
|  | t-test | -5 |  | 53 |  | -4 |  | 52 |  | -3 |  | 53 |
|  | MMRM-CS | 0 |  | 68 |  | -1 |  | 62 |  | -1 |  | 58 |
|  | MMRM-UN | 0 |  | 68 |  | -1 |  | 62 |  | -1 |  | 58 |
| MAR with equal dropout of 40% in each group | | | | |  |  |  |  |  |  |  |  |
|  | t-test | -4 |  | 55 |  | -3 |  | 55 |  | -1 |  | 56 |
|  | MMRM-CS | 0 |  | 69 |  | -1 |  | 63 |  | 0 |  | 60 |
|  | MMRM-UN | 0 |  | 69 |  | 0 |  | 63 |  | 0 |  | 60 |
| MNAR with unequal dropout of 30% and 50% in each group, one reason | | | | | | | | |  |  |  |  |
|  | t-test | -17 |  | 42 |  | -16 |  | 43 |  | -15 |  | 44 |
|  | MMRM-CS | -6 |  | 64 |  | -9 |  | 55 |  | -12 |  | 50 |
|  | MMRM-UN | -6 |  | 64 |  | -9 |  | 56 |  | -12 |  | 50 |
| MNAR with unequal dropout of 30% and 50% in each group, two reasons | | | | | | | | |  |  |  |  |
|  | t-test | -8 |  | 51 |  | -8 |  | 50 |  | -7 |  | 51 |
|  | MMRM-CS | -2 |  | 67 |  | -4 |  | 59 |  | -5 |  | 55 |
|  | MMRM-UN | -2 |  | 67 |  | -4 |  | 60 |  | -5 |  | 55 |
| MNAR with equal dropout of 40% in each group | | | | |  |  |  |  |  |  |  |  |
|  | t-test | -5 |  | 54 |  | -5 |  | 53 |  | -4 |  | 54 |
|  | MMRM-CS | -2 |  | 68 |  | -3 |  | 61 |  | -3 |  | 58 |
|  | MMRM-UN | -2 |  | 69 |  | -3 |  | 61 |  | -3 |  | 58 |

^1^ No missing data

^2^ Independent two sample t-test for the difference in group means at the final time point

^3^ Mixed model for repeated measures, compound symmetric variance-covariance matrix

^4^ Mixed model for repeated measures, unstructured variance-covariance matrix

**Supplemental Table 2. Bias and bias percent: simulation results for linear trajectory**

| **rho** | **Data** | **Method** | **Mean Bias** | **Mean Bias %** | **Min Bias** | **Min Bias %** | **Max Bias** | **Max Bias %** |
| --- | --- | --- | --- | --- | --- | --- | --- | --- |
| 0.7 | Complete | t-test | 0.007 | 0 | -5.734 | -137 | 6.031 | 144 |
| 0.7 | Complete | MMRM-CS | 0.007 | 0 | -5.734 | -137 | 6.031 | 144 |
| 0.7 | Complete | MMRM-UN | 0.007 | 0 | -5.734 | -137 | 6.031 | 144 |
| 0.7 | MCAR - equal dropout | t-test | -0.001 | 0 | -7.077 | -169 | 7.802 | 187 |
| 0.7 | MCAR - equal dropout | MMRM-CS | 0.011 | 0 | -6.604 | -158 | 6.775 | 162 |
| 0.7 | MCAR - equal dropout | MMRM-UN | 0.010 | 0 | -6.633 | -159 | 6.758 | 162 |
| 0.7 | MAR - unequal dropout, one reason | t-test | -0.624 | -15 | -8.734 | -209 | 7.429 | 178 |
| 0.7 | MAR - unequal dropout, one reason | MMRM-CS | 0.017 | 0 | -7.408 | -177 | 6.361 | 152 |
| 0.7 | MAR - unequal dropout, one reason | MMRM-UN | 0.017 | 0 | -7.356 | -176 | 6.388 | 153 |
| 0.7 | MAR - unequal dropout, different reasons | t-test | -0.190 | -5 | -8.308 | -199 | 6.880 | 165 |
| 0.7 | MAR - unequal dropout, different reasons | MMRM-CS | 0.008 | 0 | -6.463 | -155 | 6.571 | 157 |
| 0.7 | MAR - unequal dropout, different reasons | MMRM-UN | 0.006 | 0 | -6.552 | -157 | 6.424 | 154 |
| 0.7 | MAR - equal dropout | t-test | -0.045 | -1 | -8.319 | -199 | 8.099 | 194 |
| 0.7 | MAR - equal dropout | MMRM-CS | 0.018 | 0 | -6.820 | -163 | 6.600 | 158 |
| 0.7 | MAR - equal dropout | MMRM-UN | 0.017 | 0 | -6.803 | -163 | 6.746 | 161 |
| 0.7 | MNAR - unequal dropout, one reason | t-test | -0.742 | -18 | -9.534 | -228 | 6.677 | 160 |
| 0.7 | MNAR - unequal dropout, one reason | MMRM-CS | -0.250 | -6 | -7.252 | -173 | 5.813 | 139 |
| 0.7 | MNAR - unequal dropout, one reason | MMRM-UN | -0.250 | -6 | -7.373 | -176 | 5.962 | 143 |
| 0.7 | MNAR - unequal dropout, different reasons | t-test | -0.278 | -7 | -7.684 | -184 | 6.881 | 165 |
| 0.7 | MNAR - unequal dropout, different reasons | MMRM-CS | -0.084 | -2 | -6.671 | -160 | 6.013 | 144 |
| 0.7 | MNAR - unequal dropout, different reasons | MMRM-UN | -0.086 | -2 | -6.726 | -161 | 6.054 | 145 |
| 0.7 | MNAR - equal dropout | t-test | -0.142 | -3 | -7.347 | -176 | 7.070 | 169 |
| 0.7 | MNAR - equal dropout | MMRM-CS | -0.039 | -1 | -7.493 | -179 | 6.932 | 166 |
| 0.7 | MNAR - equal dropout | MMRM-UN | -0.041 | -1 | -7.447 | -178 | 6.848 | 164 |
| 0.5 | Complete | t-test | 0.005 | 0 | -5.449 | -130 | 5.929 | 142 |
| 0.5 | Complete | MMRM-CS | 0.005 | 0 | -5.449 | -130 | 5.929 | 142 |
| 0.5 | Complete | MMRM-UN | 0.005 | 0 | -5.449 | -130 | 5.929 | 142 |
| 0.5 | MCAR - equal dropout | t-test | -0.002 | 0 | -6.965 | -167 | 7.175 | 172 |
| 0.5 | MCAR - equal dropout | MMRM-CS | 0.008 | 0 | -6.680 | -160 | 6.693 | 160 |
| 0.5 | MCAR - equal dropout | MMRM-UN | 0.009 | 0 | -6.688 | -160 | 6.758 | 162 |
| 0.5 | MAR - unequal dropout, one reason | t-test | -0.447 | -11 | -7.481 | -179 | 7.123 | 170 |
| 0.5 | MAR - unequal dropout, one reason | MMRM-CS | 0.007 | 0 | -6.745 | -161 | 7.791 | 186 |
| 0.5 | MAR - unequal dropout, one reason | MMRM-UN | 0.007 | 0 | -6.709 | -161 | 7.740 | 185 |
| 0.5 | MAR - unequal dropout, different reasons | t-test | -0.142 | -3 | -7.123 | -170 | 7.439 | 178 |
| 0.5 | MAR - unequal dropout, different reasons | MMRM-CS | 0.000 | 0 | -6.783 | -162 | 7.145 | 171 |
| 0.5 | MAR - unequal dropout, different reasons | MMRM-UN | -0.003 | 0 | -6.766 | -162 | 7.279 | 174 |
| 0.5 | MAR - equal dropout | t-test | -0.017 | 0 | -7.159 | -171 | 8.701 | 208 |
| 0.5 | MAR - equal dropout | MMRM-CS | 0.019 | 0 | -6.783 | -162 | 8.332 | 199 |
| 0.5 | MAR - equal dropout | MMRM-UN | 0.020 | 0 | -6.748 | -161 | 8.332 | 199 |
| 0.5 | MNAR - unequal dropout, one reason | t-test | -0.677 | -16 | -8.730 | -209 | 7.479 | 179 |
| 0.5 | MNAR - unequal dropout, one reason | MMRM-CS | -0.382 | -9 | -7.103 | -170 | 6.320 | 151 |
| 0.5 | MNAR - unequal dropout, one reason | MMRM-UN | -0.382 | -9 | -7.092 | -170 | 6.304 | 151 |
| 0.5 | MNAR - unequal dropout, different reasons | t-test | -0.271 | -6 | -8.799 | -211 | 7.640 | 183 |
| 0.5 | MNAR - unequal dropout, different reasons | MMRM-CS | -0.154 | -4 | -7.875 | -188 | 6.437 | 154 |
| 0.5 | MNAR - unequal dropout, different reasons | MMRM-UN | -0.159 | -4 | -8.016 | -192 | 6.358 | 152 |
| 0.5 | MNAR - equal dropout | t-test | -0.131 | -3 | -7.589 | -182 | 6.795 | 163 |
| 0.5 | MNAR - equal dropout | MMRM-CS | -0.077 | -2 | -6.691 | -160 | 6.525 | 156 |
| 0.5 | MNAR - equal dropout | MMRM-UN | -0.078 | -2 | -6.622 | -158 | 6.348 | 152 |
| 0.3 | Complete | t-test | -0.002 | 0 | -5.822 | -139 | 5.968 | 143 |
| 0.3 | Complete | MMRM-CS | -0.002 | 0 | -5.822 | -139 | 5.968 | 143 |
| 0.3 | complete | MMRM-UN | -0.002 | 0 | -5.822 | -139 | 5.968 | 143 |
| 0.3 | MCAR - equal dropout | t-test | -0.006 | 0 | -7.466 | -179 | 7.426 | 178 |
| 0.3 | MCAR - equal dropout | MMRM-CS | -0.003 | 0 | -6.923 | -166 | 7.150 | 171 |
| 0.3 | MCAR - equal dropout | MMRM-UN | -0.002 | 0 | -6.880 | -165 | 6.968 | 167 |
| 0.3 | MAR - unequal dropout, one reason | t-test | -0.267 | -6 | -7.135 | -171 | 6.981 | 167 |
| 0.3 | MAR - unequal dropout, one reason | MMRM-CS | 0.004 | 0 | -7.001 | -167 | 7.703 | 184 |
| 0.3 | MAR - unequal dropout, one reason | MMRM-UN | 0.001 | 0 | -7.041 | -168 | 7.925 | 190 |
| 0.3 | MAR - unequal dropout, different reasons | t-test | -0.081 | -2 | -7.575 | -181 | 8.539 | 204 |
| 0.3 | MAR - unequal dropout, different reasons | MMRM-CS | 0.008 | 0 | -7.277 | -174 | 7.377 | 176 |
| 0.3 | MAR - unequal dropout, different reasons | MMRM-UN | 0.008 | 0 | -7.385 | -177 | 7.058 | 169 |
| 0.3 | MAR - equal dropout | t-test | -0.045 | -1 | -6.933 | -166 | 7.433 | 178 |
| 0.3 | MAR - equal dropout | MMRM-CS | -0.014 | 0 | -6.756 | -162 | 7.211 | 173 |
| 0.3 | MAR - equal dropout | MMRM-UN | -0.015 | 0 | -7.299 | -175 | 7.194 | 172 |
| 0.3 | MNAR - unequal dropout, one reason | t-test | -0.647 | -15 | -7.914 | -189 | 7.412 | 177 |
| 0.3 | MNAR - unequal dropout, one reason | MMRM-CS | -0.524 | -13 | -7.613 | -182 | 7.149 | 171 |
| 0.3 | MNAR - unequal dropout, one reason | MMRM-UN | -0.523 | -13 | -7.638 | -183 | 7.092 | 170 |
| 0.3 | MNAR - unequal dropout, different reasons | t-test | -0.254 | -6 | -7.164 | -171 | 7.737 | 185 |
| 0.3 | MNAR - unequal dropout, different reasons | MMRM-CS | -0.205 | -5 | -7.541 | -180 | 7.351 | 176 |
| 0.3 | MNAR - unequal dropout, different reasons | MMRM-UN | -0.204 | -5 | -7.542 | -180 | 7.665 | 183 |
| 0.3 | MNAR - equal dropout | t-test | -0.133 | -3 | -7.898 | -189 | 8.425 | 202 |
| 0.3 | MNAR - equal dropout | MMRM-CS | -0.103 | -2 | -7.404 | -177 | 7.741 | 185 |
| 0.3 | MNAR - equal dropout | MMRM-UN | -0.103 | -2 | -7.432 | -178 | 7.668 | 183 |

**Supplemental Table 3. Bias and bias percent: simulation results for non-linear trajectory**

| **rho** | **Data** | **Method** | **Mean Bias** | **Mean Bias %** | **Min Bias** | **Min Bias %** | **Max Bias** | **Max Bias %** |
| --- | --- | --- | --- | --- | --- | --- | --- | --- |
| 0.7 | Complete | t-test | -0.006 | 0 | -5.712 | -137 | 5.678 | 136 |
| 0.7 | Complete | MMRM-CS | -0.006 | 0 | -5.712 | -137 | 5.678 | 136 |
| 0.7 | Complete | MMRM-UN | -0.006 | 0 | -5.712 | -137 | 5.678 | 136 |
| 0.7 | MCAR - equal dropout | t-test | -0.021 | 0 | -7.358 | -176 | 7.140 | 171 |
| 0.7 | MCAR - equal dropout | MMRM-CS | -0.008 | 0 | -6.671 | -160 | 6.325 | 151 |
| 0.7 | MCAR - equal dropout | MMRM-UN | -0.009 | 0 | -6.702 | -160 | 6.362 | 152 |
| 0.7 | MAR - unequal dropout, one reason | t-test | -0.627 | -15 | -7.829 | -187 | 6.595 | 158 |
| 0.7 | MAR - unequal dropout, one reason | MMRM-CS | -0.019 | 0 | -6.413 | -153 | 6.901 | 165 |
| 0.7 | MAR - unequal dropout, one reason | MMRM-UN | -0.019 | 0 | -6.575 | -157 | 6.982 | 167 |
| 0.7 | MAR - unequal dropout, different reasons | t-test | -0.225 | -5 | -7.943 | -190 | 7.804 | 187 |
| 0.7 | MAR - unequal dropout, different reasons | MMRM-CS | -0.002 | 0 | -5.972 | -143 | 6.450 | 154 |
| 0.7 | MAR - unequal dropout, different reasons | MMRM-UN | -0.004 | 0 | -5.995 | -143 | 6.348 | 152 |
| 0.7 | MAR - equal dropout | t-test | -0.154 | -4 | -8.908 | -213 | 7.598 | 182 |
| 0.7 | MAR - equal dropout | MMRM-CS | -0.011 | 0 | -6.514 | -156 | 6.786 | 162 |
| 0.7 | MAR - equal dropout | MMRM-UN | -0.012 | 0 | -6.740 | -161 | 6.744 | 161 |
| 0.7 | MNAR - unequal dropout, one reason | t-test | -0.723 | -17 | -8.903 | -213 | 6.980 | 167 |
| 0.7 | MNAR - unequal dropout, one reason | MMRM-CS | -0.259 | -6 | -6.125 | -147 | 6.765 | 162 |
| 0.7 | MNAR - unequal dropout, one reason | MMRM-UN | -0.259 | -6 | -6.174 | -148 | 6.712 | 161 |
| 0.7 | MNAR - unequal dropout, different reasons | t-test | -0.314 | -8 | -7.188 | -172 | 6.629 | 159 |
| 0.7 | MNAR - unequal dropout, different reasons | MMRM-CS | -0.091 | -2 | -6.433 | -154 | 6.214 | 149 |
| 0.7 | MNAR - unequal dropout, different reasons | MMRM-UN | -0.092 | -2 | -6.303 | -151 | 6.182 | 148 |
| 0.7 | MNAR - equal dropout | t-test | -0.215 | -5 | -7.105 | -170 | 7.908 | 189 |
| 0.7 | MNAR - equal dropout | MMRM-CS | -0.066 | -2 | -6.366 | -152 | 6.370 | 152 |
| 0.7 | MNAR - equal dropout | MMRM-UN | -0.068 | -2 | -6.398 | -153 | 6.410 | 153 |
| 0.5 | Complete | t-test | -0.019 | 0 | -5.830 | -139 | 5.399 | 129 |
| 0.5 | Complete | MMRM-CS | -0.019 | 0 | -5.830 | -139 | 5.399 | 129 |
| 0.5 | Complete | MMRM-UN | -0.019 | 0 | -5.830 | -139 | 5.399 | 129 |
| 0.5 | MCAR - equal dropout | t-test | -0.010 | 0 | -7.378 | -177 | 7.434 | 178 |
| 0.5 | MCAR - equal dropout | MMRM-CS | -0.004 | 0 | -6.548 | -157 | 7.032 | 168 |
| 0.5 | MCAR - equal dropout | MMRM-UN | -0.004 | 0 | -6.385 | -153 | 7.061 | 169 |
| 0.5 | MAR - unequal dropout, one reason | t-test | -0.441 | -11 | -8.171 | -195 | 6.951 | 166 |
| 0.5 | MAR - unequal dropout, one reason | MMRM-CS | -0.009 | 0 | -6.774 | -162 | 6.634 | 159 |
| 0.5 | MAR - unequal dropout, one reason | MMRM-UN | -0.011 | 0 | -6.812 | -163 | 6.576 | 157 |
| 0.5 | MAR - unequal dropout, different reasons | t-test | -0.180 | -4 | -7.118 | -170 | 6.863 | 164 |
| 0.5 | MAR - unequal dropout, different reasons | MMRM-CS | -0.026 | -1 | -7.251 | -173 | 7.105 | 170 |
| 0.5 | MAR - unequal dropout, different reasons | MMRM-UN | -0.025 | -1 | -7.018 | -168 | 7.069 | 169 |
| 0.5 | MAR - equal dropout | t-test | -0.122 | -3 | -7.499 | -179 | 6.937 | 166 |
| 0.5 | MAR - equal dropout | MMRM-CS | -0.021 | -1 | -7.129 | -171 | 6.256 | 150 |
| 0.5 | MAR - equal dropout | MMRM-UN | -0.020 | 0 | -7.273 | -174 | 6.228 | 149 |
| 0.5 | MNAR - unequal dropout, one reason | t-test | -0.671 | -16 | -7.758 | -186 | 6.668 | 160 |
| 0.5 | MNAR - unequal dropout, one reason | MMRM-CS | -0.395 | -9 | -6.899 | -165 | 7.216 | 173 |
| 0.5 | MNAR - unequal dropout, one reason | MMRM-UN | -0.393 | -9 | -6.945 | -166 | 7.066 | 169 |
| 0.5 | MNAR - unequal dropout, different reasons | t-test | -0.322 | -8 | -8.799 | -211 | 6.653 | 159 |
| 0.5 | MNAR - unequal dropout, different reasons | MMRM-CS | -0.182 | -4 | -7.875 | -188 | 6.385 | 153 |
| 0.5 | MNAR - unequal dropout, different reasons | MMRM-UN | -0.181 | -4 | -8.016 | -192 | 6.499 | 155 |
| 0.5 | MNAR - equal dropout | t-test | -0.222 | -5 | -7.395 | -177 | 7.178 | 172 |
| 0.5 | MNAR - equal dropout | MMRM-CS | -0.132 | -3 | -6.925 | -166 | 6.763 | 162 |
| 0.5 | MNAR - equal dropout | MMRM-UN | -0.132 | -3 | -6.878 | -165 | 6.818 | 163 |
| 0.3 | Complete | t-test | -0.009 | 0 | -5.880 | -141 | 5.454 | 130 |
| 0.3 | Complete | MMRM-CS | -0.009 | 0 | -5.880 | -141 | 5.454 | 130 |
| 0.3 | complete | MMRM-UN | -0.009 | 0 | -5.880 | -141 | 5.454 | 130 |
| 0.3 | MCAR - equal dropout | t-test | -0.031 | -1 | -7.701 | -184 | 6.844 | 164 |
| 0.3 | MCAR - equal dropout | MMRM-CS | -0.028 | -1 | -7.982 | -191 | 6.837 | 164 |
| 0.3 | MCAR - equal dropout | MMRM-UN | -0.027 | -1 | -7.949 | -190 | 6.904 | 165 |
| 0.3 | MAR - unequal dropout, one reason | t-test | -0.275 | -7 | -8.573 | -205 | 6.827 | 163 |
| 0.3 | MAR - unequal dropout, one reason | MMRM-CS | -0.009 | 0 | -8.269 | -198 | 7.425 | 178 |
| 0.3 | MAR - unequal dropout, one reason | MMRM-UN | -0.010 | 0 | -8.073 | -193 | 7.409 | 177 |
| 0.3 | MAR - unequal dropout, different reasons | t-test | -0.114 | -3 | -7.304 | -175 | 7.130 | 171 |
| 0.3 | MAR - unequal dropout, different reasons | MMRM-CS | -0.022 | -1 | -6.930 | -166 | 6.753 | 162 |
| 0.3 | MAR - unequal dropout, different reasons | MMRM-UN | -0.021 | -1 | -7.064 | -169 | 6.877 | 165 |
| 0.3 | MAR - equal dropout | t-test | -0.062 | -1 | -8.886 | -213 | 6.448 | 154 |
| 0.3 | MAR - equal dropout | MMRM-CS | 0.002 | 0 | -8.119 | -194 | 6.663 | 159 |
| 0.3 | MAR - equal dropout | MMRM-UN | 0.002 | 0 | -7.979 | -191 | 6.546 | 157 |
| 0.3 | MNAR - unequal dropout, one reason | t-test | -0.632 | -15 | -8.466 | -203 | 6.416 | 154 |
| 0.3 | MNAR - unequal dropout, one reason | MMRM-CS | -0.512 | -12 | -8.151 | -195 | 6.288 | 150 |
| 0.3 | MNAR - unequal dropout, one reason | MMRM-UN | -0.513 | -12 | -8.172 | -196 | 6.285 | 150 |
| 0.3 | MNAR - unequal dropout, different reasons | t-test | -0.276 | -7 | -8.013 | -192 | 6.967 | 167 |
| 0.3 | MNAR - unequal dropout, different reasons | MMRM-CS | -0.219 | -5 | -8.504 | -203 | 6.739 | 161 |
| 0.3 | MNAR - unequal dropout, different reasons | MMRM-UN | -0.218 | -5 | -8.496 | -203 | 6.713 | 161 |
| 0.3 | MNAR - equal dropout | t-test | -0.181 | -4 | -7.016 | -168 | 7.540 | 180 |
| 0.3 | MNAR - equal dropout | MMRM-CS | -0.136 | -3 | -6.542 | -157 | 7.466 | 179 |
| 0.3 | MNAR - equal dropout | MMRM-UN | -0.137 | -3 | -6.692 | -160 | 7.435 | 178 |

**Supplemental Table 4. Comparison of t-test, mixed model for repeated measures with compound symmetric variance-covariance, and mixed model for repeated measures with unstructured variance-covariance, with respect to bias percent and power; simulation results for linear trajectory with limited (10-15%) missing data**

|  |  | ρ=0.7 | |  | ρ=0.5 | |  | ρ=0.3 | |
| --- | --- | --- | --- | --- | --- | --- | --- | --- | --- |
|  |  | (N=10,000) | |  | (N=10,000) | |  | (N=10,000) | |
|  |  | Bias % | Power |  | Bias % | Power |  | Bias % | Power |
| Complete | |  |  |  |  |  |  |  |  |
|  | t-test | 0 | 80 |  | 0 | 80 |  | 0 | 80 |
|  | MMRM-CS | 0 | 80 |  | 0 | 81 |  | 0 | 80 |
|  | MMRM-UN | 0 | 80 |  | 0 | 80 |  | 0 | 80 |
| MCAR with equal dropout of 10% in each group | | | | | |  |  |  |  |
|  | t-test | 0 | 76 |  | 0 | 76 |  | 0 | 75 |
|  | MMRM-CS | 0 | 78 |  | 0 | 78 |  | 0 | 76 |
|  | MMRM-UN | 0 | 78 |  | 0 | 77 |  | 0 | 76 |
| MAR with unequal dropout of 10% and 15% in each group, one reason | | | | | | | | | |
|  | t-test | -2 | 73 |  | -2 | 73 |  | -1 | 73 |
|  | MMRM-CS | 0 | 78 |  | 0 | 77 |  | 0 | 75 |
|  | MMRM-UN | 0 | 78 |  | 0 | 77 |  | 0 | 75 |
| MNAR with unequal dropout of 10% and 15% in each group, one reason | | | | | | | | | |
|  | t-test | -2 | 72 |  | -2 | 73 |  | -2 | 73 |
|  | MMRM-CS | -1 | 77 |  | -1 | 75 |  | -1 | 74 |
|  | MMRM-UN | -1 | 77 |  | -1 | 75 |  | -1 | 74 |
