# Supplementary material for: Single time point comparisons in longitudinal randomized controlled trials: power and bias in the presence of missing data
Source: BMC Med Res Methodol. 2016 Apr 12;16:43. doi: 10.1186/s12874-016-0144-0 (PMC4828848; doi:10.1186/s12874-016-0144-0)
Supplement: Additional file 2: — Supplement SAS simulation and analysis ELA20160310. (PDF 17 kb) [file 12874_2016_144_MOESM2_ESM.pdf]

```
*evaluate the performance of the t-test and MMRM in terms of bias and power;

libname dbox 'c:\simulations\results';

%let true = 4.18;

%macro readresults(data);

data Tmeans_&data&vare; set dbox.Tmeans_&data&vare; run;

data Tpvalues_&data&vare; set dbox.Tpvalues_&data&vare; run;

data MMRMCS_&data&vare; set dbox.MMRMCS_&data&vare; run;

data MMRMUN_&data&vare; set dbox.MMRMUN_&data&vare; run;


*calculate bias;
data tbias;
    set Tmeans_&data&vare;
    if class='Diff (1-2)';
    bias = (-1*mean - &true);*estimated mean diff - true difference in means;
    biaspct = ((-1*mean - &true)/&true) *100;*percent bias;
run;
*report bias;
title "&data&vare - t-test BIAS";
proc means data=tbias n min p25 median p75 max mean std maxdec=4;
    var bias biaspct;
    output out=tablet&data&vare;
run;
*indicator var for reject H0 at 0.05 significance;
data tpower;
   set Tpvalues_&data&vare;
   if method='Pooled';
   RejectH0 = (Probt < 0.05);*H0: same mean Y for treatment and control groups at timepoint 4;
run;
*report power: proportion: (# that reject H0)/NumSamples;
```

```
title "&data&vare - t-test POWER";
proc freq data=tpower;
   tables RejectH0 / nocum;
run;
***********************************************************************;
**MM CS;
data mmcsresult;
    set MMRMCS_&data&vare;
    bias = (estimate - &true);
    biaspct = ((estimate - &true)/&true) *100;
    RejectH0 = (Probt < 0.05);
run;
title "&data&vare - MM CS BIAS";
proc means data=mmcsresult n min p25 median p75 max mean std maxdec=4;
    var bias biaspct;
    output out=tablemmcs&data&vare;
run;
title "&data&vare - MM CS POWER";
proc freq data=mmcsresult;
   tables RejectH0 / nocum;
run;

**MM UN;
data mmunresult;
    set MMRMUN_&data&vare;
    bias = (estimate - &true);
    biaspct = ((estimate - &true)/&true) *100;
    RejectH0 = (Probt < 0.05);
run;
title "&data&vare - MM UN BIAS";
proc means data=mmunresult n min p25 median p75 max mean std maxdec=4;
    var bias biaspct;
    output out=tablemmun&data&vare;
run;
title "&data&vare - MM UN POWER";
proc freq data=mmunresult;
   tables RejectH0 / nocum;
run;
```

```
data tablebias&data&vare;
    length sim $10;
    set tablet&data&vare(in=t)
        tablemmcs&data&vare(in=cs)
        tablemmun&data&vare(in=un);
    if t then method='t-test ';
    else if cs then method='MMRM-CS';
    else if un then method='MMRM-UN';

    sim="&data";
    vare="&vare";
run;

proc append base=table force data=tablebias&data&vare; run;
%mend;

%macro maketable(vare);
    %let vare = &vare;

    %readresults(wide);

    %readresults(wideMCAR);

    %readresults(wideMARa4);

    %readresults(wideMARb4);

    %readresults(wideMAR34);

    %readresults(wideMNARa);

    %readresults(wideMNARb);

    %readresults(wideMNAR5);
%mend;

ods html close;
```

```
ods html;
title ' ';
%maketable(33);*within-person variance (rho=.7);
%maketable(55);*within-person variance (rho=.5);
%maketable(77);*within-person variance (rho=.3);


*run all three values of rho first;
*then table the results with the code below;


proc sort data=table; by vare sim method; run;
data tableit(keep=order1 order2 sim method vare meanbias meanbiaspct minbias minbiaspct maxbias maxbiaspct);
    length order1 order2 8 vare $2;
    do until(last.method);
        set table;
        by vare sim method;

        if sim='wide'    then order1=   1   ;
        else if sim='wideMCAR'   then order1=   2   ;
        else if sim='wideMARa4'  then order1=   3   ;
        else if sim='wideMARb4'  then order1=   4   ;
        else if sim='wideMAR34'  then order1=   5   ;
        else if sim='wideMNARa'  then order1=   6   ;
        else if sim='wideMNARb'  then order1=   7   ;
        else if sim='wideMNAR5'  then order1=   8   ;

        if method='t-test' then order2=1;
        else if method='MMRM-CS' then order2=2;
        else if method='MMRM-UN' then order2=3;

        if _stat_='MEAN' then do;
            meanbias=bias;
            meanbiaspct=biaspct;
        end;
        else if _stat_='MIN' then do;
            minbias=bias;
            minbiaspct=biaspct;
```

```
        end;
        else if _stat_='MAX' then do;
            maxbias=bias;
            maxbiaspct=biaspct;
        end;
    end;
run;
proc sort data=tableit; by vare order1 order2; run;

data dbox.tableit;
    set tableit;
run;
```
