# Supplementary material for: Single time point comparisons in longitudinal randomized controlled trials: power and bias in the presence of missing data
Source: BMC Med Res Methodol. 2016 Apr 12;16:43. doi: 10.1186/s12874-016-0144-0 (PMC4828848; doi:10.1186/s12874-016-0144-0)
Supplement: Additional file 3: — Supplement SAS evaluate performance ELA20160310. (PDF 24 kb) [file 12874_2016_144_MOESM3_ESM.pdf]

```
*simulate repeated measures data;
*compare bias and power of t-test and means model;
*single time point comparison;
*ELA April 2, 2015;

libname dbox 'c:\simulations\results';

%let n = 200;*sample size;
%let NumSamples = 10000;*number of simulated data sets;

*** rho = .7 ****************************;
%let varb = 77;*between-person variance;
%let vare = 33;*within-person variance;
*****************************************;
/*** rho = .5 ****************************;
%let varb = 55;*between-person variance;
%let vare = 55;*within-person variance;
*****************************************/;
/*** rho = .3 ****************************;
%let varb = 33;*between-person variance;
%let vare = 77;*within-person variance;
*****************************************/;

%macro ODSOff(); /* Call prior to BY-group processing */
ods graphics off;
ods exclude all;
ods noresults;
%mend;

%macro ODSOn(); /* Call after BY-group processing */
ods graphics on;
ods exclude none;
ods results;
%mend;


********************************************************************************;
*** SIMULATE COMPLETE DATA *****************************************************;
```

```
********************************************************************************;
*one row per person;
data wide;
    call streaminit(20090520);
    do SampleID =1 to &NumSamples;
        do id=1 to &n;
            b = rand('Normal',0, sqrt(&varb));*between-person effects;
            if mod(id,2)=0 then do;
                trt = 0; *control group;
                do until((y1>=0 & y1<=100));
                    y1 = rand('Normal',50, sqrt(&vare)) + b;
                end;
                do until((y2>=0 & y2<=100));
                    y2 = rand('Normal',50, sqrt(&vare)) + b;
                end;
                do until((y3>=0 & y3<=100));
                    y3 = rand('Normal',50, sqrt(&vare)) + b;
                end;
                do until((y4>=0 & y4<=100));
                    y4 = rand('Normal',50, sqrt(&vare)) + b;
                end;
            end;
            else do;
                trt = 1; *active treatment group;
                do until((y1>=0 & y1<=100));
                    y1 = rand('Normal',50, sqrt(&vare)) + b;
                end;
                do until((y2>=0 & y2<=100));
                    y2 = rand('Normal',51.393, sqrt(&vare)) + b;
                end;
                do until((y3>=0 & y3<=100));
                    y3 = rand('Normal',52.7867, sqrt(&vare)) + b;
                end;
                do until((y4>=0 & y4<=100));
                    y4 = rand('Normal',54.18, sqrt(&vare)) + b;
                end;
            end;
            output;
```

```
        end;
    end;
run;

*********************************************************************************;
*** MCAR with equal dropout of 40% in each group ******************************;
*********************************************************************************;
proc sort data=wide; by sampleid trt; run;
proc surveyselect data=wide
    method=SRS n=40 seed=2015 out=mcar;
    strata sampleid trt;
run;
proc sort data=mcar; by sampleid id; run;
proc sort data=wide; by sampleid id; run;

data wideMCAR;
    merge   wide
            mcar(in=m keep=sampleid id);
    by sampleid id;
    call streaminit(2015);
    if m then do;
        U = rand('Uniform');
        if U < 1/3 then do;
            y2=.; y3=.; y4=.;
        end;
        else if U < 2/3 then do;
            y3=.; y4=.;
        end;
        else if U < 1 then do;
            y4=.;
        end;
    end;
run;
*********************************************************************************;
*** 2. MAR with unequal dropout of 30% and 50% in each group **********************;
*** participants in the treatment group have a dropout rate of 30%;
*** participants in the control group have a dropout rate of 50%;
*** a) One reason for dropout: feeling poorly (low score);
```

```
*********************************************************************************;
*set probability of missing in control arm;
%let TRT0PMT2=.15;
%let TRT0PMT3=.3;
%let TRT0PMT4=.5;
*set probability of missing in treatment arm;
%let TRT1PMT2=.1;
%let TRT1PMT3=.2;
%let TRT1PMT4=.3;

data wideMARa2;
    set wide;
    call streaminit(2015);

    EY1=50;*expectation for Y at time 1;

    if trt=0 then p2 = ((100*&TRT0PMT2)/(100-EY1))*(100 - y1)/100;*probability of missing at t2 depends on y at t1;
    else if trt=1 then p2 = ((100*&TRT1PMT2)/(100-EY1))*(100 - y1)/100;

    m2 = rand("Bernoulli", p2);
    if m2=1 then do; y2=.; y3=.; y4=.; end;*dropout at t2;
run;

*calculate the mean of y2 now in each arm;
proc sql noprint;
   select mean(y2) into :EY2trt0 - :EY2trt1 from widemara2
   group by trt;
quit;
%put _user_;

data widemara3;
    set widemara2;
    if m2=0 then do;
        if trt = 0 then p3 = (1 - (1-&TRT0PMT3)/(1-&TRT0PMT2))*(100/(100-&EY2trt0)) * (100 - y2)/100;*probability of mis
        else if trt = 1 then p3 = (1 - (1-&TRT1PMT3)/(1-&TRT1PMT2))*(100/(100-&EY2trt1)) * (100 - y2)/100;
        m3 = rand("Bernoulli", p3);
        if m3=1 then do; y3=.; y4=.; end;*dropout at t3;
    end;
```

```
run;

*calculate the mean of y3 now in each arm;
proc sql noprint;
   select mean(y3) into :EY3trt0 - :EY3trt1 from widemara3
   group by trt;
quit;
%put _user_;

data widemara4;
    set widemara3;
    if m3 =0 then do;
        if trt = 0 then p4 = (1 - (1- &TRT0PMT4)/(1- &TRT0PMT3))*(100/(100- &EY3trt0)) * (100 - y3)/100;*probability of
        else if trt =1 then p4 = (1 - (1- &TRT1PMT4)/(1- &TRT1PMT3))*(100/(100- &EY3trt1)) * (100 - y3)/100;
        m4 = rand("Bernoulli", p4);
        if m4=1 then do; y4=.; end;*dropout at t4;
    end;
run;

***************************************************************************************;
********************************************************************************;
*** 2. MAR with unequal dropout of 30% and 50% in each group *********************;
*** participants in the treatment group have a dropout rate of 30%;
*** participants in the control group have a dropout rate of 50%;
*** b) Different reasons for dropout: feeling poorly (low score) or feeling well (high score);
********************************************************************************;
*set probability of missing in control arm;
%let TRT0PMT2=.15;
%let TRT0PMT3=.3;
%let TRT0PMT4=.5;
*set probability of missing in treatment arm;
%let TRT1PMT2=.1;
%let TRT1PMT3=.2;
%let TRT1PMT4=.3;

data wideMARb2;
    set wide;
    call streaminit(2015);
```

```
    EY1=50;*expectation for Y at time 1;

    if trt=0 then c = &TRT0PMT2 / (.6 * (((55-EY1)/55)**2) + .4);
    else if trt=1 then c = &TRT1PMT2 / (.6 * (((55-EY1)/55)**2) + .4);

    p2 = c * (.6 * (((55-y1)/55)**2) + .4);*probabilty of missing at t2 depends on y1;

    m2 = rand("Bernoulli", p2);
    if m2=1 then do; y2=.; y3=.; y4=.; end;*dropout at t2;
run;

*calculate the mean of y2 now in each arm;
proc sql noprint;
   select mean(y2) into :EY2trt0 - :EY2trt1 from widemarb2
   group by trt;
quit;
%put _user_;

data widemarb3;
    set widemarb2;
    if m2=0 then do;

        if trt=0 then c = (1 - (1 - &TRT0PMT3)/(1 - &TRT0PMT2)) / (.6 * (((55-&EY2trt0)/55)**2) + .4);
        else if trt=1 then c = (1 - (1 - &TRT1PMT3)/(1 - &TRT1PMT2)) / (.6 * (((55-&EY2trt1)/55)**2) + .4);

        p3 = c * (.6 * (((55-y2)/55)**2) + .4);*probabilty of missing at t3 depends on y2;

        m3 = rand("Bernoulli", p3);
        if m3=1 then do; y3=.; y4=.; end;*dropout at t3;
    end;
run;

*calculate the mean of y3 now in each arm;
proc sql noprint;
   select mean(y3) into :EY3trt0 - :EY3trt1 from widemarb3
   group by trt;
quit;
```

```
%put _user_;

data widemarb4;
    set widemarb3;
    if m3 =0 then do;
        if trt=0 then c = (1 - (1 - &TRT0PMT4)/(1 - &TRT0PMT3)) / (.6 * (((55-&EY3trt0)/55)**2) + .4);
        else if trt=1 then c = (1 - (1 - &TRT1PMT4)/(1 - &TRT1PMT3)) / (.6 * (((55-&EY3trt1)/55)**2) + .4);

        p4 = c * (.6 * (((55-y3)/55)**2) + .4);*probabilty of missing at t4 depends on y3;

        m4 = rand("Bernoulli", p4);
        if m4=1 then do; y4=.; end;*dropout at t4;
    end;
run;

*************************************************************************************;
*************************************************************************************;
*** 3. MAR with equal dropout of 40% in each group *********************************;
***  Different reasons for dropout: feeling poorly (low score) or feeling well (high score);
**************************************************************************************;
*set probability of missing in control arm;
%let TRT0PMT2=.15;
%let TRT0PMT3=.25;
%let TRT0PMT4=.4;
*set probability of missing in treatment arm;
%let TRT1PMT2=.15;
%let TRT1PMT3=.25;
%let TRT1PMT4=.4;
data wideMAR32;
    set wide;
    call streaminit(2015);

    EY1=50;*expectation for Y at time 1;

    if trt=0 then c = &TRT0PMT2 / (.6 * (((55-EY1)/55)**2) + .4);
    else if trt=1 then c = &TRT1PMT2 / (.6 * (((55-EY1)/55)**2) + .4);

    p2 = c * (.6 * (((55-y1)/55)**2) + .4);*probabilty of missing at t2 depends on y1;

    m2 = rand("Bernoulli", p2);
    if m2=1 then do; y2=.; y3=.; y4=.; end;*dropout at t2;
run;

*calculate the mean of y2 now in each arm;
proc sql noprint;
   select mean(y2) into :EY2trt0 - :EY2trt1 from widemar32
   group by trt;
quit;
%put _user_;

data widemar33;
    set widemar32;
    if m2=0 then do;

        if trt=0 then c = (1 - (1 - &TRT0PMT3)/(1 - &TRT0PMT2)) / (.6 * (((55-&EY2trt0)/55)**2) + .4);
        else if trt=1 then c = (1 - (1 - &TRT1PMT3)/(1 - &TRT1PMT2)) / (.6 * (((55-&EY2trt1)/55)**2) + .4);

        p3 = c * (.6 * (((55-y2)/55)**2) + .4);*probabilty of missing at t3 depends on y2;

        m3 = rand("Bernoulli", p3);
        if m3=1 then do; y3=.; y4=.; end;*dropout at t3;
    end;
run;

*calculate the mean of y3 now in each arm;
proc sql noprint;
   select mean(y3) into :EY3trt0 - :EY3trt1 from widemar33
   group by trt;
quit;
%put _user_;

data widemar34;
    set widemar33;
    if m3 =0 then do;
        if trt=0 then c = (1 - (1 - &TRT0PMT4)/(1 - &TRT0PMT3)) / (.6 * (((55-&EY3trt0)/55)**2) + .4);
        else if trt=1 then c = (1 - (1 - &TRT1PMT4)/(1 - &TRT1PMT3)) / (.6 * (((55-&EY3trt1)/55)**2) + .4);
```

```
        p4 = c * (.6 * (((55-y3)/55)**2) + .4);*probabilty of missing at t4 depends on y3;

        m4 = rand("Bernoulli", p4);
        if m4=1 then do; y4=.; end;*dropout at t4;
    end;
run;

********************************************************************************;
*** 4. MNAR with unequal dropout of 30% and 50% in each group **********************;
*** participants in the treatment group have a dropout rate of 30%;
*** participants in the control group have a dropout rate of 50%;
*** a) One reason for dropout: feeling poorly (low score);
********************************************************************************;
*set probability of missing in control arm;
%let TRT0PMT2=.15;
%let TRT0PMT3=.3;
%let TRT0PMT4=.5;
*set probability of missing in treatment arm;
%let TRT1PMT2=.1;
%let TRT1PMT3=.2;
%let TRT1PMT4=.3;

data wideMNARa;
    set wide;
    call streaminit(2015);

    EY2trt0=50;*expectation for Y at time 2 in the control group;
    EY3trt0=50;
    EY4trt0=50;

    EY2trt1=51.393;*expectation for Y at time 2 in the treatment group;
    EY3trt1=52.7867;
    EY4trt1=54.18;

    if trt=0 then p2 = ((100*&TRT0PMT2)/(100-EY2trt0))*(100 - y2)/100;*probability of missing at t2 depends on y at t2;
    else if trt=1 then p2 = ((100*&TRT1PMT2)/(100-EY2trt1))*(100 - y2)/100;
```

```
    m2 = rand("Bernoulli", p2);
    if m2=1 then do; y2=.; y3=.; y4=.; end;*dropout at t2;
    else do;
        if trt=0 then p3=(1 - (1-&TRT0PMT3)/(1-&TRT0PMT2))*(100/(100-EY3trt0))*(100-y3)/100;
        else if trt=1 then p3=(1 - (1-&TRT1PMT3)/(1-&TRT1PMT2))*(100/(100-EY3trt1))*(100-y3)/100;

        m3 = rand("Bernoulli", p3);
        if m3=1 then do; y3=.; y4=.; end;*dropout at t3;
        else do;
            if trt=0 then p4=(1 - (1-&TRT0PMT4)/(1-&TRT0PMT3))*(100/(100-EY4trt0))*(100-y4)/100;
            else if trt=1 then p4=(1 - (1-&TRT1PMT4)/(1-&TRT1PMT3))*(100/(100-EY4trt1))*(100-y4)/100;

            m4 = rand("Bernoulli", p4);
            if m4=1 then do; y4=.; end;*dropout at t4;
        end;
    end;
run;

***************************************************************************************;
********************************************************************************;
*** 4. MNAR with unequal dropout of 30% and 50% in each group **********************;
*** participants in the treatment group have a dropout rate of 30%;
*** participants in the control group have a dropout rate of 50%;
*** b) Different reasons for dropout: feeling poorly (low score) or feeling well (high score);
********************************************************************************;
*set probability of missing in control arm;
%let TRT0PMT2=.15;
%let TRT0PMT3=.3;
%let TRT0PMT4=.5;
*set probability of missing in treatment arm;
%let TRT1PMT2=.1;
%let TRT1PMT3=.2;
%let TRT1PMT4=.3;

data wideMNARb;
    set wide;
    call streaminit(2015);
```

```
    EY2trt0=50;*expectation for Y at time 2 in the control group;
    EY3trt0=50;
    EY4trt0=50;

    EY2trt1=51.393;*expectation for Y at time 2 in the treatment group;
    EY3trt1=52.7867;
    EY4trt1=54.18;

    if trt=0 then c = &TRT0PMT2 / (.6 * (((55-EY2trt0)/55)**2) + .4);
    else if trt=1 then c = &TRT1PMT2 / (.6 * (((55-EY2trt1)/55)**2) + .4);

    p2 = c * (.6 * (((55-y2)/55)**2) + .4);*probabilty of missing at t2 depends on y2;

    m2 = rand("Bernoulli", p2);
    if m2=1 then do; y2=.; y3=.; y4=.; end;*dropout at t3;
    else do;
        if trt=0 then c = (1 - (1 - &TRT0PMT3)/(1 - &TRT0PMT2)) / (.6 * (((55-EY3trt0)/55)**2) + .4);
        else if trt=1 then c = (1 - (1 - &TRT1PMT3)/(1 - &TRT1PMT2)) / (.6 * (((55-EY3trt1)/55)**2) + .4);

        p3 = c * (.6 * (((55-y3)/55)**2) + .4);*probabilty of missing at t3 depends on y3;

        m3 = rand("Bernoulli", p3);
        if m3=1 then do; y3=.; y4=.; end;*dropout at t3;
        else do;
            if trt=0 then c = (1 - (1 - &TRT0PMT4)/(1 - &TRT0PMT3)) / (.6 * (((55-EY4trt0)/55)**2) + .4);
            else if trt=1 then c = (1 - (1 - &TRT1PMT4)/(1 - &TRT1PMT3)) / (.6 * (((55-EY4trt1)/55)**2) + .4);

            p4 = c * (.6 * (((55-y4)/55)**2) + .4);*probabilty of missing at t4 depends on y4;

            m4 = rand("Bernoulli", p4);
            if m4=1 then do; y4=.; end;*dropout at t4;
        end;
    end;
run;


*********************************************************************************;
*** 5. MNAR with equal dropout of 40% in each group **********************;
```

```
*** Different reasons for dropout: feeling poorly (low score) or feeling well (high score);
*********************************************************************************;
*set probability of missing in control arm;
%let TRT0PMT2=.15;
%let TRT0PMT3=.25;
%let TRT0PMT4=.4;
*set probability of missing in treatment arm;
%let TRT1PMT2=.15;
%let TRT1PMT3=.25;
%let TRT1PMT4=.4;

data wideMNAR5;
    set wide;
    call streaminit(2015);

    EY2trt0=50;*expectation for Y at time 2 in the control group;
    EY3trt0=50;
    EY4trt0=50;

    EY2trt1=51.393;*expectation for Y at time 2 in the treatment group;
    EY3trt1=52.7867;
    EY4trt1=54.18;

    if trt=0 then c = &TRT0PMT2 / (.6 * (((55-EY2trt0)/55)**2) + .4);
    else if trt=1 then c = &TRT1PMT2 / (.6 * (((55-EY2trt1)/55)**2) + .4);

    p2 = c * (.6 * (((55-y2)/55)**2) + .4);*probabilty of missing at t2 depends on y2;

    m2 = rand("Bernoulli", p2);
    if m2=1 then do; y2=.; y3=.; y4=.; end;*dropout at t3;
    else do;
        if trt=0 then c = (1 - (1 - &TRT0PMT3)/(1 - &TRT0PMT2)) / (.6 * (((55-EY3trt0)/55)**2) + .4);
        else if trt=1 then c = (1 - (1 - &TRT1PMT3)/(1 - &TRT1PMT2)) / (.6 * (((55-EY3trt1)/55)**2) + .4);

        p3 = c * (.6 * (((55-y3)/55)**2) + .4);*probabilty of missing at t3 depends on y3;

        m3 = rand("Bernoulli", p3);
        if m3=1 then do; y3=.; y4=.; end;*dropout at t3;
```

```
        else do;
            if trt=0 then c = (1 - (1 - &TRT0PMT4)/(1 - &TRT0PMT3)) / (.6 * (((55-EY4trt0)/55)**2) + .4);
            else if trt=1 then c = (1 - (1 - &TRT1PMT4)/(1 - &TRT1PMT3)) / (.6 * (((55-EY4trt1)/55)**2) + .4);

            p4 = c * (.6 * (((55-y4)/55)**2) + .4);*probabilty of missing at t4 depends on y4;

            m4 = rand("Bernoulli", p4);
            if m4=1 then do; y4=.; end;*dropout at t4;
        end;
    end;
run;



*****************************************************************************************;
*** DATA ANALYSIS OF SIMULATED DATA *****************************************************;
*****************************************************************************************;
%macro analysis(data);
*one row per person PER TIMEPOINT;
data long;
    set &data;*read in wide data;

    time = 1; y = y1; output;
    time = 2; y = y2; output;
    time = 3; y = y3; output;
    time = 4; y = y4; output;
    drop y1 y2 y3 y4 b;
run;

*** t-test bias and power **************************************************************;
proc ttest data=long;
    by SampleId;
    where time=4; *only consider last timepoint;
    class trt;
    var Y;
    ods output ttests=Tpvalues statistics=Tmeans;
run;
```

```
options nonotes;

*** MMRM with Compound Symmetric variance-covariance matrix assumed ****;
proc mixed data=long;
      by SampleID;
      class id trt time;
      model y = trt time trt*time / noint;
      repeated time / type=CS subject=id;
      estimate 'MM t4' trt -1 1 trt*time 0 0 0 -1 0 0 0 1/cl ;
      ODS OUTPUT estimates = MMRMCS;
run;

*** MMRM with unstructured variance-covariance matrix ******************;
proc mixed data=long;
      by SampleID;
      class id trt time;
      model y = trt time trt*time / noint;
      repeated time / type=UN subject=id;
      estimate 'MM t4' trt -1 1 trt*time 0 0 0 -1 0 0 0 1/cl ;
      ODS OUTPUT estimates = MMRMUN;
run;
options notes;


*** output the results to dropbox **************************************;
data dbox.Tpvalues_&data&vare; set Tpvalues; run;

data dbox.Tmeans_&data&vare; set Tmeans; run;

data dbox.MMRMCS_&data&vare; set MMRMCS; run;

data dbox.MMRMUN_&data&vare; set MMRMUN; run;
**********************************************************************;
%mend;

%ODSOff;

%analysis(wide);
```

```
%analysis(wideMCAR);

%analysis(wideMARa4);

%analysis(wideMARb4);

%analysis(wideMAR34);

%analysis(wideMNARa);

%analysis(wideMNARb);

%analysis(wideMNAR5);

%ODSon;
```
